# Supplementary material for: Serum and urinary biomarkers for early detection of acute kidney injury following Hypnale spp. envenoming
Source: PLoS Negl Trop Dis. 2021 Dec 6;15(12):e0010011. doi: 10.1371/journal.pntd.0010011 (PMC8675918; doi:10.1371/journal.pntd.0010011)
Supplement: S1 Table — *Correlation coefficients and p values given for No AKI vs AKI 2–3. (DOCX) [file pntd.0010011.s001.docx]

**S1 Table.** Peak biomarker concentration reached within 24h post-bite. Absolute biomarker concentrations are presented as medians and interquartile ranges (No AKI, AKI stage 1 and AKI stage 2-3 were compared and correlated using Kendall’s tau-b test with single tailed p-value)

*Correlation coefficients and p values given for No AKI vs AKI 2-3

| **Biomarker** | **No AKI** | **AKI 1** | **AKI 2-3** | **Correlation coefficient*** | **p value*** |
| --- | --- | --- | --- | --- | --- |
| sCr (mg/dL) | 0.86 (0.76-0.96) | 1.30 (1.20-1.30) | 3.1 (1.90-4.0) | 0.600 | 0.071 |
| sCysC (mg/L) | 1.0 (0.85-1.6) | 0.95 (0.77-1.4) | 4.9 (1.8-5.9) | 0.333 | 0.301 |
| uNGAL (ng/ml) | 32 (17-48) | 29 (17-166) | 761 (29-3054) | 0.333 | 0.301 |
| uCysC (ng/ml) | 32 (15-62) | 9 (6-1485) | 5013 (620-8241) | 0.183 | 0.359 |
| uβ2M (ng/ml) | 75 (20-456) | 322 (60-723) | 3197 (790-8947) | -0.333 | 0.248 |
| uClu (ng/ml) | 59 (18-95) | 17 (8-1388) | 1112 (32-5712) | 0.333 | 0.301 |
| uAlb (ng/ml) | 10543 (3060-22377) | 14671(539-176740) | 60621(6667-291331) | 0.333 | 0.248 |
| uTFF3 (ng/ml) | 607 (334-1531) | 2358(710-8063) | 1471 (786-8165) | 0.333 | 0.248 |
| uKIM1 (ng/ml) | 0.41 (0.18-0.76) | 0.42 (0.18-2.7) | 0.26 (0.14-0.37) | 0.333 | 0.301 |
| uOPN (ng/ml) | 515 (365-1065) | 894 (232-2215) | 826 (311-3241) | 0.667 | 0.087 |
