# Supplementary figures and images for: Serum and urinary biomarkers for early detection of acute kidney injury following Hypnale spp. envenoming
Source: PLoS Negl Trop Dis. 2021 Dec 6;15(12):e0010011. doi: 10.1371/journal.pntd.0010011 (PMC8675918; doi:10.1371/journal.pntd.0010011)

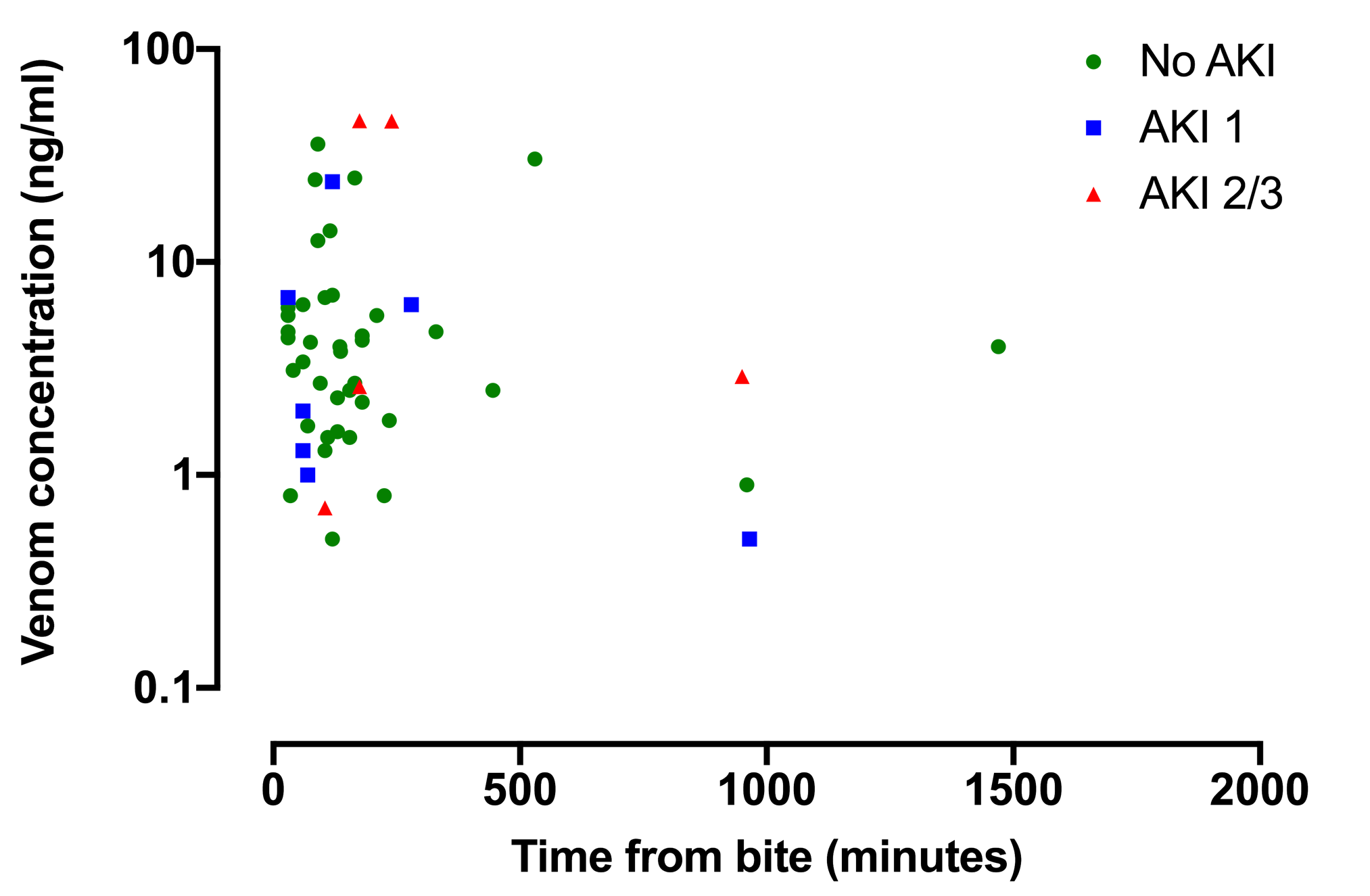

Supplement: S1 Fig — (TIF) [file pntd.0010011.s002.tif]

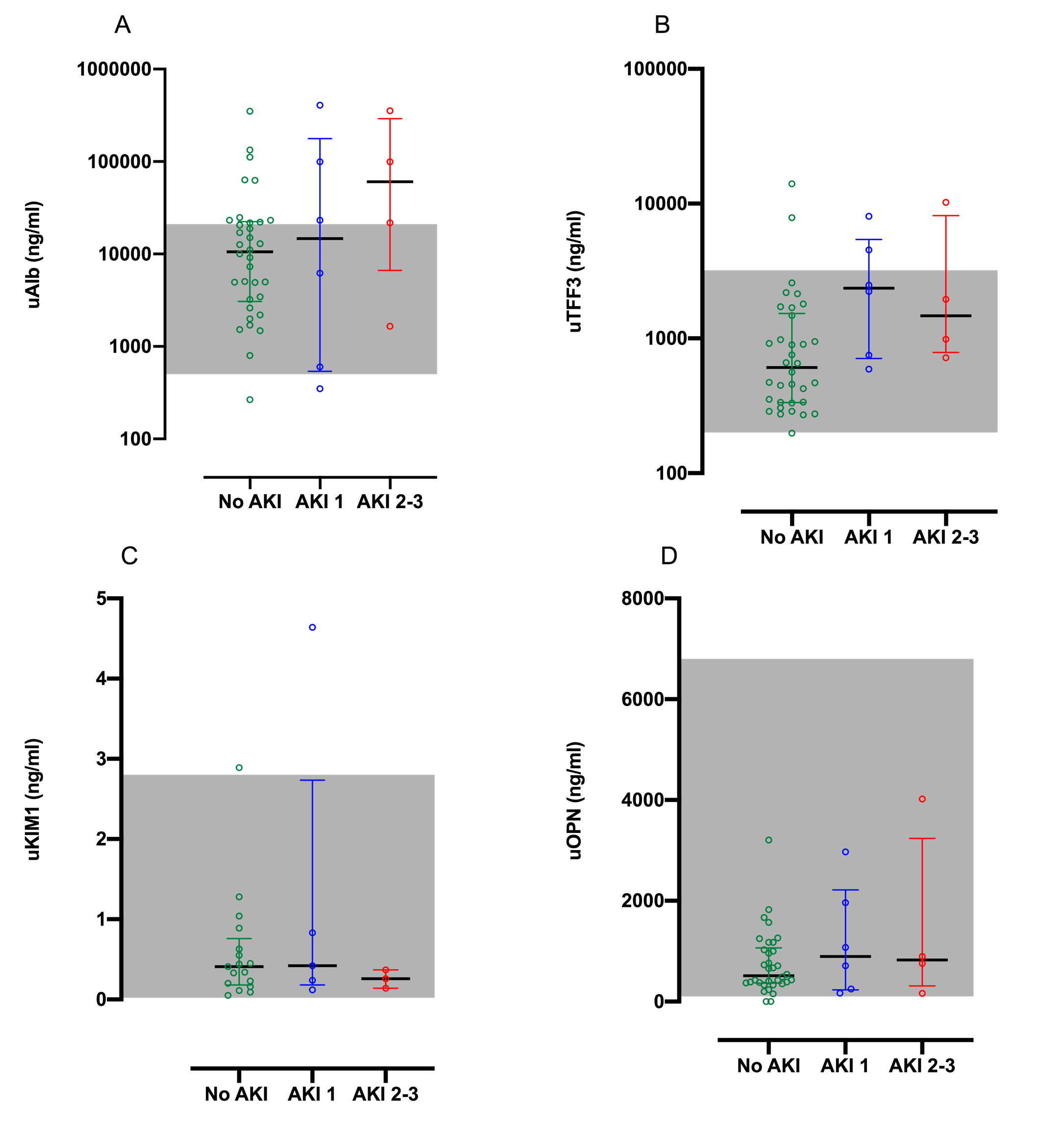

Supplement: S2 Fig — Maximum renal biomarker concentration within 24 h of bite. Scatter plots of the maximum biomarker concentrations reached within 24 h post-bite for patients with no acute kidney injury (No AKI; green), mild AKI (AKI 1; blue) and moderate to severe AKI (AKI 2–3; red), for urinary albumin (uAlb; Panel A), urinary trefoil factor-3 (uTFF3; Panel B), urinary kidney injury molecule-1 (uKIM-1; Panel C) and urinary osteopontin (uOPN; Panel D). The grey shaded is the normal range based on respective biomarkers measured in healthy individuals. (TIF) [file pntd.0010011.s003.tif]

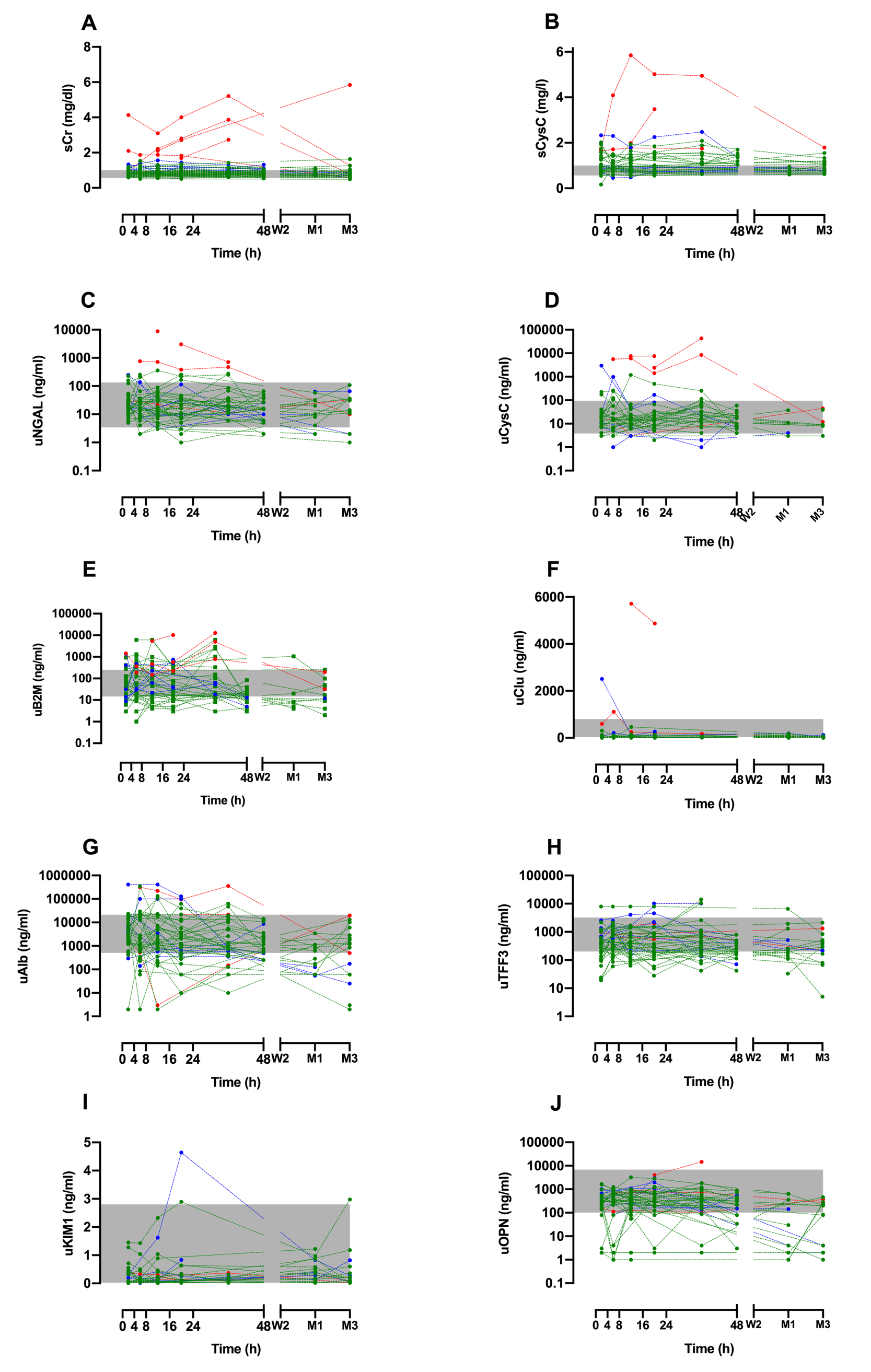

Supplement: S3 Fig — Patients without AKI ([No AKI; green], with AKI stage 1 [Mild AKI; blue] and with AKI stages 2–3 [moderate/severe AKI; red]). serum creatinine (sCr; Panel A), serum cystatin C (sCysC; Panel B), urinary neutrophil gelatinase associated lipocalin (uNGAL; Panel C), urinary cystatin C (uCysC; Panel D), urinary beta-2 microglobulin (uβ2M; Panel E), urinary clusterin (uClu; Panel F), urinary albumin (uAlb; Panel G), urinary trefoil factor-3 (uTFF3; Panel H), urinary kidney injury molecule-1 (uKIM-1; Panel I) and urinary osteopontin (uOPN; Panel J). The grey shaded is the normal range based on respective biomarkers measured in healthy individuals. (TIF) [file pntd.0010011.s004.tif]

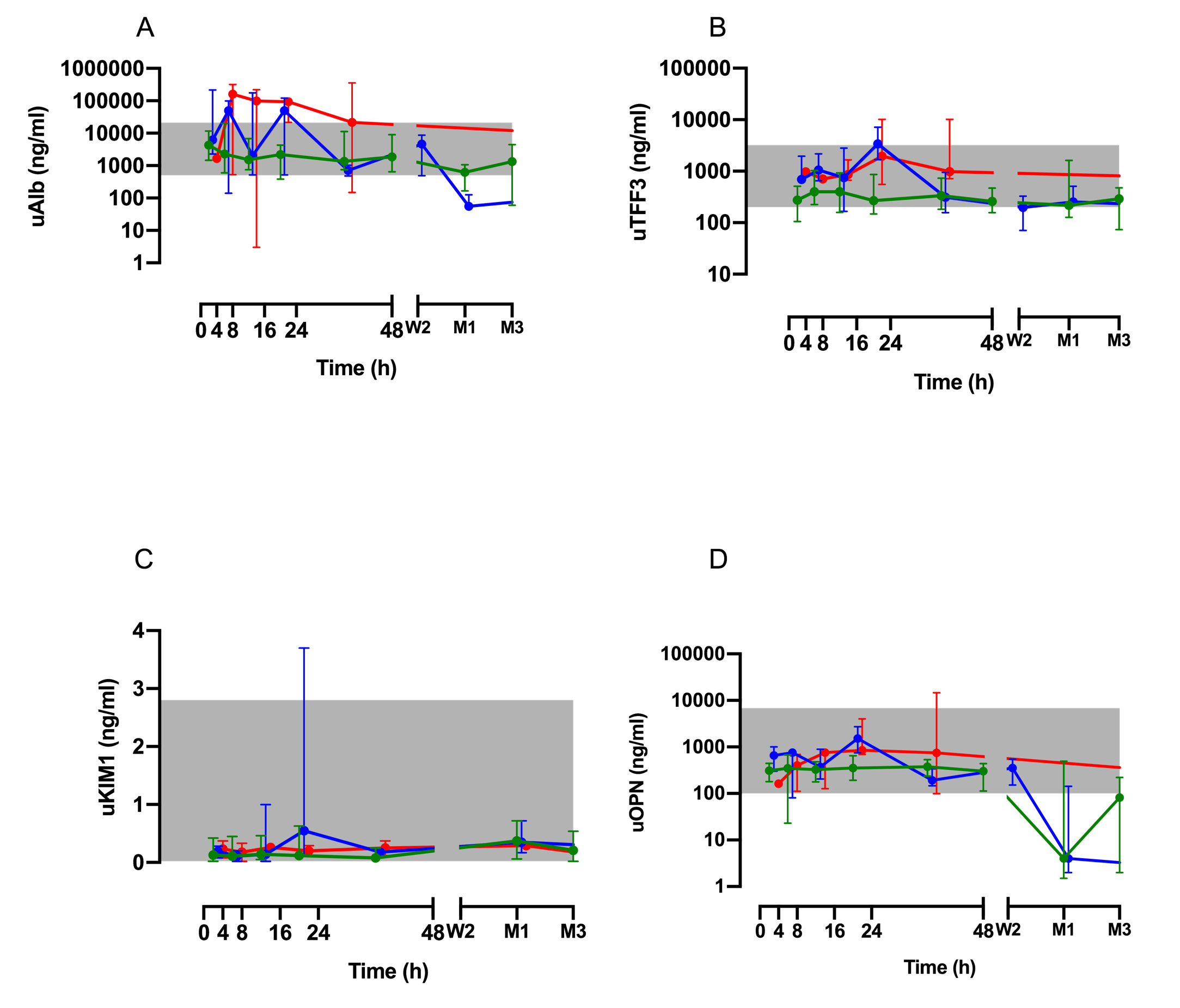

Supplement: S4 Fig — Time course of the median biomarker concentrations (with interquartile ranges) for each of the three patient groups ([No AKI; green], [Mild AKI; blue] and [moderate to severe AKI; red]) post-bite for 48 h including urinary albumin (uAlb; Panel A), urinary trefoil factor-3 (uTFF3; Panel B), urinary kidney injury molecule-1 (uKIM-1; Panel C) and urinary osteopontin (uOPN; Panel D). The grey shaded is the normal range based on respective biomarkers measured in healthy individuals. (TIF) [file pntd.0010011.s005.tif]
