# Supplementary material for: Serum and urinary biomarkers for early detection of acute kidney injury following Hypnale spp. envenoming
Source: PLoS Negl Trop Dis. 2021 Dec 6;15(12):e0010011. doi: 10.1371/journal.pntd.0010011 (PMC8675918; doi:10.1371/journal.pntd.0010011)
Supplement: Attachment — Submitted filename: Review_PNTD-D-21-01041R1.pdf [file pntd.0010011.s008.pdf]

November 11th, 2021

Dear Editor,

This is the second round of revision for the manuscript PNTD-D-21-01041R1 entitle “Serum and urinary biomarkers for early detection of acute kidney injury following Hypnale spp. Envenoming”.

The main weakness of this study is the very small sample size and the fact that the authors included an experimental group of other study about antivenom therapy. The issue with the small sample size was raised by all the reviewers and the author’s response was that this fact was “added as a limitation of the study in the discussion” (the sentence “This was added as a limitation of the study in the discussion (Page 31) appear <u>7 times</u> in the Responses to reviewers’ letter). Regarding other questions raised by the reviewers such as about venom mechanisms, validity of new AKI biomarkers, new sample analysis, increasing sample size by grouping all cases of AKI together… for the most of these questions the author’s limited to respond that “…this was not the focus of the study”; “… is not routinely performed”; “… were not measured in the present study”; “… increasing the sample size is beyond the scope of the current study”; “… this study is not aimed to assess the causality of AKI”. When questioned by the reviewer 2 about what was the novel biomarkers for kidney injury detection in patients with HNV envenoming… the author’s limited to respond “We agree with the reviewer that further studies are required to understand the precise mechanism of venom that induce kidney injury and look specifically for the novel biomarkers responding to that mechanism. We also agree that serum Cystatin C may not be a specific marker for AKI in HNV envenoming but simply a marker of functional AKI in this setting, similar to AKI that occurs with other types of snakes, such as Russell’s viper”. So… what was the novelty here? Creatinine and Cystatin C are biomarkers of almost all types of kidney injury. The reviewer 3 mentioned that “the most critical point of the work is the sample size of the AKI 2-3 group, composed of only 5 patients. With this population size, the authors were not able to perform any statistical significance between groups, which makes the results only suggestive and not conclusive. “As a major weakness of the study, the population size of moderate/severe AKI patients did not provided enough credibility to the study”. The author’s response was: “We agree that this is suggestive but not conclusive”.

I really agree that venom-induced AKI is an important health problem to be addressed and high-quality epidemiological studies are needed mainly in neglected areas. However, in my opinion, PNTD is a traditional high-impact journal that is not suitable for preliminary or suggestive studies. So, my recommendation is to reject.
