# Supplementary material for: Serum and urinary biomarkers for early detection of acute kidney injury following Hypnale spp. envenoming
Source: PLoS Negl Trop Dis. 2021 Dec 6;15(12):e0010011. doi: 10.1371/journal.pntd.0010011 (PMC8675918; doi:10.1371/journal.pntd.0010011)
Supplement: Attachment — Submitted filename: PLOS NTD review.docx [file pntd.0010011.s006.docx]

August 19^th^, 2021

To the Editor in Chief

PLoS Neglected Tropical Diseases

Manuscript number: **PNTD-D-21-01041**

Manuscript title: **Serum and urinary biomarkers for early detection of acute kidney injury following** *Hypnale* **spp. Envenoming.**

**Comments to the Editor:**

In this manuscript Wijewickrama and colleagues conducted a clinical study to evaluate the feasibility of classical kidney injury biomarkers for the detection of acute kidney injury (AKI) severity in patients envenomed by the hump-nosed pit viper (*Hypnale* spp). *Hypnale* spp snakes are one of the five most important venomous snakes in Sri Lanka and AKI is the main clinical manifestation of systemic envenoming. Its main conclusion is that serum cystatin C was the best marker in the earlier period of envenomation, while serum creatine performs best in later phases, which surprisingly differ from literature data that indicate NGAL as a good marker in case of other snake envenomations.

This work presents a good panel of tested biomarkers in different time-points with a good clinical sample size for this type of outcome. Certainly, it has valuable epidemiological significance mainly addressing a local problem of envenomation in Sri Lanka. The main weakness is the 23 of 52 (44%) of patients eligible and included for biomarker tests were also participants of a polyspecific antivenom clinical trial, so it is not possible to discharge an interference in AKI severity evaluations, mainly during the follow-up phases of study. Another point to consider is the lack of a mechanistic discussion or data about venom components and venom-induced pathology, which is important in this case.

In summary, the authors conducted a clinical study with reasonable epidemiological data that helps to understand a local problem of envenomation in Sri Lanka, but lacks in novelty, since do not present data about the mechanism of venom-induced AKI. Thus, I don’t know if this manuscript is suitable for the general audience of PLoS NTD or fits better in a more specific journal. Bellow you can find my comments and suggestions to the authors that may help you in the final decision.

**Comments to the author:**

This study presents a clinical evaluation of several classical kidney injury biomarkers for the detection of acute kidney injury (AKI) severity in patients envenomed by the hump-nosed pit viper (*Hypnale* spp). A range of ten different urinary and serum kidney injury biomarkers were measured in different time-points including in follow up of two weeks, one and three months-post envenomation. A reasonable sample size was included for this type of outcome. Certainly, it has valuable epidemiological significance mainly addressing a local problem of envenomation in Sri Lanka.

Bellow is described major and minor points that the authors should consider for review.

*Major points:*

1. The major weakness of this study seems to be the fact that 23 of 52 (44%) of patients eligible and included for biomarker tests were also participants of other study that evaluates a polyspecific antivenom in a clinical trial. Thus, it is possible that some of these patients had received antivenom therapy and probably this event has some interference in AKI severity evaluations, mainly during the later follow-up phases of study. The authors only mention this fact in discussion section (page 33). I think this fact must be included and detailed in methods section. The data analysis for this group of patients also needs to be considered as an independent group.

2. Little is mentioned by the authors about venom composition, its mechanisms of action, possible mechanism of renal injury etc… I think it would be interesting to include more information about that.

3. Since the authors have access to clinical samples (serum and urine) in different time-points for the different severity groups why not include also a panel of serum/urine biochemistry and hematological data?

4. Since heme released from hemoglobin/myoglobin is an important event to be considered in venom-induced nephrotoxicity, did the authors check if patients have myo/hemoglobinuria? How about hemoglobin serum levels? Hemolysis is a common event in *Hypnale* spp envenomation?

5. How about the imaging exams from these patients? This kind of data also would be interesting to include, since the authors probably have access to clinical data.

6. The median venom serum concentration detected in envenomed victims seems not correlate with AKI severity. How did the authors explain this data? How about the venom clearance by the kidney? Urinary venom concentration was measured? There is some direct nephrotoxin with action on kidney structure already identified in HNV?

7. Patients that evolved to AKI had concomitantly coagulation disturbances? It would be interesting to include some discussion about that. Triggering coagulation and contact system can cause different changes in renal physiology, like intraglomerular thrombus deposition, changes in glomerular vessel permeability, reduction in GFR, electrolyte tubular transport and changes in urinary concentration ability by collecting ducts.

8. How the authors explain the best performance of serum creatinine as a kidney injury biomarker when compared to urinary NGAL, which is a specific protein released by renal tubules upon injury? A more critical discussion and accurate data literature comparison about this result it would be interesting.

*Minor points:*

1. Please review table 1 for layout and format errors. Information of median time to admission for “AKI stage 2/3” column is incomprehensible and in different format comparing to other columns.

2. Figure 2 presents the same information about venom concentration previously showed in table 1? It was measured in serum or urine samples? Please include this information both in table 1 and figure 2 captions.

3. Reference citations seems to be out of journal format. Please double check journal rules.
