# Supplementary material for: Serum and urinary biomarkers for early detection of acute kidney injury following Hypnale spp. envenoming
Source: PLoS Negl Trop Dis. 2021 Dec 6;15(12):e0010011. doi: 10.1371/journal.pntd.0010011 (PMC8675918; doi:10.1371/journal.pntd.0010011)
Supplement: Attachment — Submitted filename: Responses to reviewers commnets.docx [file pntd.0010011.s007.docx]

Responses to reviewers’ comments

| Reviewer’s comment | Response |
| --- | --- |
| Reviewer 1 |  |
| Methods |  |
| 1. The major weakness of this study seems to be the fact that 23 of 52 (44%) of patients eligible and included for biomarker tests were also participants of other study that evaluates a polyspecific antivenom in a clinical trial. Thus, it is possible that some of these patients had received antivenom therapy and probably this event has some interference in AKI severity evaluations, mainly during the later follow-up phases of study. The authors only mention this fact in discussion section (page 33). I think this fact must be included and detailed in methods section. The data analysis for this group of patients also needs to be considered as an independent group. | Thank you. The use of experimental antivenom in some envenomed patients was added to the methods section (Page 8). Number of patients receiving the experimental antivenom is already stated in the footnote of table 1.  Incidence of AKI was 2/22 (9%) in the group that received the experimental antivenom. In comparison 8/26 (31%) developed AKI in the group that did not receive the experimental antivenom. 4 patients in whom the data on the use of antivenom was not available were excluded from this analysis. The small overall sample size and specifically, the small numbers with AKI, make meaningful statistical comparisons on the effect of the experimental antivenom in the development of AKI and the levels of biomarkers between the two groups difficult.  These observations were added to the results section (Page 15) and further discussed in the discussion section under limitations (Page 30). |
| Results |  |
| 3. Since the authors have access to clinical samples (serum and urine) in different time-points for the different severity groups why not include also a panel of serum/urine biochemistry and hematological data? | The research proposal was developed to obtain data on AKI and biomarkers in serum and urine. Serum creatinine has been included. Haematological data requires different samples. A full blood count requires an EDTA tube, and coagulation studies require a citrated tube – neither of these were collected. This information has been previously published in other cohorts. |
| 4. Since heme released from hemoglobin/myoglobin is an important event to be considered in venom-induced nephrotoxicity, did the authors check if patients have myo/hemoglobinuria? How about hemoglobin serum levels? Hemolysis is a common event in *Hypnale* spp envenomation? | We agree that rhabdomyolysis and intravascular haemolysis, especially in the setting of microangiopathic haemolytic anaemia, could contribute to the development of AKI in *Hypnale* spp. envenoming. However, the focus of the study was on early diagnosis of AKI rather than an investigation of the pathophysiology of thrombotic microangiopathy. Therefore, we did not assess myo/hemoglobinuria and hemoglobin levels were not done routinely in the patients and EDTA tubes were not collected for the study. |
| 5. How about the imaging exams from these patients? This kind of data also would be interesting to include, since the authors probably have access to clinical data. | Ultrasound scan of the kidneys would be helpful in the assessment of AKI, especially in excluding obstructive uropathy. However, this is not routinely performed in snakebite related AKI considering that it is likely to be caused by direct or indirect effects of the venom. |
| Conclusions |  |
| 2. Little is mentioned by the authors about venom composition, its mechanisms of action, possible mechanism of renal injury etc… I think it would be interesting to include more information about that. | A paragraph on venom composition, toxicity and possible mechanism of AKI in *Hypnale* spp. envenoming added to the discussion section of the manuscript (Pages 29-30). |
| 6. The median venom serum concentration detected in envenomed victims seems not correlate with AKI severity. How did the authors explain this data? How about the venom clearance by the kidney? Urinary venom concentration was measured? There is some direct nephrotoxin with action on kidney structure already identified in HNV? | Venom concentration detected is dependent on many variables including the time of presentation post- bite (Supplementary Fig. 1). Furthermore, the lack of correlation between venom concentrations and the severity of AKI suggests the existence venom concentration-independent mechanisms in the causation of AKI in *Hypnale* envenoming. This explanation was added to the discussion section (Pages 29-30).  Urinary venom concentrations are difficult to measure accurately and require timed urine samples with volume measurements. Therefore, urine venom concentrations were not measured in the present study.  A direct nephrotoxin affecting the kidney structure and function in the setting of HNV bite has not yet been elucidated. |
| 7. Patients that evolved to AKI had concomitantly coagulation disturbances? It would be interesting to include some discussion about that. Triggering coagulation and contact system can cause different changes in renal physiology, like intraglomerular thrombus deposition, changes in glomerular vessel permeability, reduction in GFR, electrolyte tubular transport and changes in urinary concentration ability by collecting ducts. | The reviewer is correct that AKI in snakebite appears to be a complication of thrombotic microangiopathy, which is associated with venom induced consumption coagulopathy. The venom induced consumption coagulopathy in HNV envenoming is mild, and requires comprehensive clotting studies (INR, fibrinogen, D-Dimer) to be detected. These were not available in this study as discussed above. The reviewer is correct that there are recognized pathological changes in the kidneys due to the thrombotic microangiopathy, but no renal biopsy data was available in this study. |
| 8. How the authors explain the best performance of serum creatinine as a kidney injury biomarker when compared to urinary NGAL, which is a specific protein released by renal tubules upon injury? A more critical discussion and accurate data literature comparison about this result it would be interesting. | The diagnosis of AKI and the severity of AKI was graded using serum creatinine in this study (based on KDIGO criteria). Unsurprisingly, this means that creatinine will perform the best overall, because it is being used as the gold standard. The aim in this study was to determine if other renal biomarkers, such as NGAL, became abnormal earlier than serum creatinine. The only way to demonstrate that NGAL, and other more specific renal biomarkers perform better, would be to have a tissue diagnosis of renal injury – which is ethically not possible. . |
| Editorial and data presentation modifications |  |
| 1. Please review table 1 for layout and format errors. Information of median time to admission for “AKI stage 2/3” column is incomprehensible and in different format comparing to other columns. | Table 1 layout and format errors corrected. Information in AKI 2-3 column modified. (Pages 14-15) |
| 2. Figure 2 presents the same information about venom concentration previously showed in table 1? It was measured in serum or urine samples? Please include this information both in table 1 and figure 2 captions. | Venom concentrations were measured in serum. This information was added to the table and figure 2 captions. (Pages 14 and 16) |
| 3. Reference citations seem to be out of journal format. Please double check journal rules. | In text reference numbers cited in square brackets |
| Summary and general comments |  |
|  |  |
| Reviewer 2 |  |
| Methods |  |
| Sample size is not sufficient for prove the hypothesis in AKI1 and AKI2-3 group. | This was added as a limitation of the study in the discussion (Page 31) |
|  |  |
| Results |  |
| Moreover, the population size in AKI stage 1 and 2-3 is too small  (n= 7 and n = 5) and should not be a proper representative population. | This was added as a limitation of the study in the discussion (Page 31) |
| Also, the biomarker scatter plot such as uCysC and uClu in AKI 1 as well as sCysC and uClu in AKI 2-3 is considerably large indicating that the patient factor might interfere the biomarker level. The author should increase the sample size in AKI group or should take individual factors into account to reduce the error of the analysis.  Suggestion: In case the sample size cannot be increased, other statistical analysis such as linear regression should be analyzed. | We agree that it would be ideal to increase the sample size and improve the statistical analysis. However, increasing the sample size is beyond the scope of the current study.  We appreciate the suggestion by the reviewer of an alternative analysis. However, the sample size is not sufficient to conduct a regression analysis as well. Furthermore, this study is not aimed to assess the causality of AKI.  When the AUC-ROC values are plotted for each of the biomarker at different time points for AKI versus no AKI sCysC does not perform as well as sCr at 0-4 h post-bite. This may be indicative of the fact that AKI 2-3 behaves as a distinct group compared to individuals with AKI 1. This is supported further by the fact that there was no difference in the peak biomarker concentrations within 24 h (Fig.3) and time dependent changes in the median biomarker concentrations (Fig. 4) between patients with no AKI and AKI 1 but a marked difference in biomarker levels in individuals with AKI 2-3.   |
| Conclusions |  |
| This manuscript proposed to investigate the novel biomarkers for kidney injury detection in patients with HNV envenoming.  The biomarkers which were chosen in this study have been thoroughly studied in previous reports despite the differences of the toxicants. The outstanding biomarkers in patients with snake envenoming in this manuscript are not different from those demonstrated in patients with other toxicants.  Suggestion: Further study should be focused and discovered the precise mechanism of venom that induce kidney injury and look specifically for the novel biomarkers responding to that mechanism. Cystatin C have been well acknowledged as a best for AKI prediction for several nephrotoxic substances so it not surprises when in HNV envenoming patients with AKI had high level of Cystatin C. This indicates that Cystatin C is not a specific marker for HNV envenoming. | We agree with the reviewer that further studies are required to understand the precise mechanism of venom that induce kidney injury and look specifically for the novel biomarkers responding to that mechanism. A statement was added to the conclusions to highlight this. (Page 32)  We also agree that serum Cystatin C may not be a specific marker for AKI in HNV envenoming but simply a marker of functional AKI in this setting, similar to AKI that occurs with other types of snakes, such as Russell’s viper. |
| Editorial and data presentation modifications |  |
| None |  |
| Summary and general comments |  |
| None |  |
| Reviewer 3 |  |
| Methods |  |
| The methods used in the present work are well described and performed properly to test the hypothesis stated. Ethical aspects were also adequate.  - The overall population size of the study was very impressive, and the number of elegible patients for the study comprehensive. However, the most critical point of the work is the sample size of the AKI 2-3 group, composed of only 5 patients.  With this population size, the authors were not able to perform any statistical significance between groups, which makes the results only suggestive and not conclusive. As a suggestion, the authors could perform the study of AKI predictive markers without classifying the event's severity (AKI patients and No AKI patients). Accepting this, the authors would have enough and conclusive data. In this case, sCysC values are found to be elevated in both AKI1 and AKI2-3 patients, where it is very likely that the prediction results for this biomarker are valid and relevant for AKI (regardless of the severity of the event). | Although we agree with the reviewer that group all cases of AKI together would increase the power of the study, visual inspection of the data strongly suggests that AKI1 and AKI2/3 are different. This is the reason that we kept these groups separate. We agree that this is suggestive but not conclusive. |
| Also, in this manner, the authors could also evaluate the difference of the development of AKI in patients submitted to the two possible antivenoms used in for the treatment. | Incidence of AKI was 2/22 (9%) in the group that received the experimental antivenom. In comparison 8/26 (31%) developed AKI in the group that did not receive the experimental antivenom. 4 patients in whom the data on the use of antivenom was not available were excluded from this analysis. Although the group receiving the experimental antivenom had a lower incidence of AKI, the small overall sample size and specifically, the small numbers with AKI, make meaningful statistical comparisons on the effect of the experimental antivenom in the development of AKI and the levels of biomarkers between the two groups difficult.  These observations were added to the results section (Page 15) and further discussed in the discussion section under limitations (Page 30). |
| For the AKI classification, the authors use a baseline value for the markers (serum or urine) “defined as the lowest serum creatinine greater than 0.4 mg/dl recorded on admission or during follow up”. It is also known that AKI cases are late events, manifesting in most cases after 48 hours. However, once the data "time to assistance" were clear to all patients (only results in table 1), there would be a possibility of a patient being admitted 24 hours after the accident (common in several regions of the world) and already having a picture of renal dysfunction being installed. Thus, baseline-based AKI classification could be compromised. Do the authors limit the length of "time to assistance" in patients' eligibility criteria? It might be interesting for the authors to inform what was the time limit for treating patients in the article. | Absence of a baseline serum creatinine is a challenge in the diagnosis AKI in any study. In order to overcome this, and specifically to address this issue of patients being admitted late with already established AKI, we used the lowest serum creatinine recorded after admission as the baseline AKI. For patients admitted late this would be the lowest serum creatinine at follow-up during recovery phase of AKI. We have documented this in the methods section.  Therefore we did not have a time limit from the bite as an inclusion criterion for the study. |
| Still in the METHODS section, the authors mention “Normal reference ranges for the serum and urinary biomarker levels were based on the findings of a previous study conducted among healthy adult volunteers.(14)". The reference is based on a previous study, where most of the authors are the same as the present study. However, these are samples quantified at different times and perhaps using different batch. As many kits are not routinely used in the laboratory, the variation between dosages can be large. Also, the difference in population group may be a biased factor. In summary, do the authors consider that this can interfere with the values determined as “normal reference ranges”? | This study was part of large cohort on AKI following self-poisoning and snake envenomation. Majority of the samples were run together to minimize any batch variation in kits.  It is common practice to use the published value in the literature as a normal range as assaying and reporting biomarkers regularly using healthy subject is uncommon. Most of the healthy patient’s demographic characteristics were similar. (Ref. Mohamed F, Buckley NA, Pickering JW, Wunnapuk K, Dissanayake S, Chathuranga U, et al. Nephrotoxicity-induced proteinuria increases biomarker diagnostic thresholds in acute kidney injury. BMC Nephrol. 2017;18(1):1–10.) |
| Results |  |
| The results are solid and have an excellent number of markers assessed, all  well suited and directly related to  AKI events. The authors noted that Cystatin C was an important biomarker in predicting a severe/moderate event within 4 hours of poisoning, giving credibility to its use in the clinical approach.  - In the RESULTS section "Peak biomarker concentrations and AKI" in Figure 3. The authors should also mention that the increased biomarkers values of moderate/servere AKI patients were also above “normal reference range” (healthy volunteers ), indicating a trust applicability in using the mentioned markers as predictors. | A statement that peak biomarker concentrations within 24 h for sCr, sCysC, uCysC and uβ2M were above the normal reference ranges was added to the results section (Page 17). |
| Also, biomarkers sCr and 2β2M were also increased in AKI I patients (compared to healthy donor), indicating that both are also valuable markers to predict AKI independently if mild,  moderate or severe. This is also valid for others results. | sCr is part of the KDIGO definition used for the diagnosis of AKI stage 1. Therefore it is expected that the peak biomarker concentration of sCr should be above the normal reference range. As for uβ2M although the median peak concentration was above the normal reference range there was a wide range making it a less valid biomarker for diagnosis of AKI stage 1. |
| Still in the same section, the authors use correlation using Kendall’s tau-b test. However, in S1 table, is not possible to determine to which comparisons the correlation coefficient and p value are (No AKI vs AKI 1 or No AKI vs AKI 2-3, AKI1 vs AKI 2-3??). | Correlation coefficients and p values are given for No AKI vs AKI 2-3. This was added to the S1 table caption. (Page 37) |
| In the RESULTS section" Long-term biomarker levels", the authors could explore better the results, showing that AKI2-3 patients presented decreased eGFR compared to others, but still (the median) within normal limits. Also, the authors focus on two patients (1 from AKI2-3 group and other from No AKI) who presented decreased levels of eGFR (CKD) and showed long term sCr values (Figure 6B). In Figure 6B, patient A (blue) was from AKI2-3 group and patient B (red) from No AKI group? | The patient A (blue) did not develop AKI. He was from no AKI group. Patient B (red) developed AKI 3. He is from the AKI 2-3 group.  To make this clearer, the colour of patient A has been changed to green in Figure 6B. (Page 26) |
| Conclusions |  |
| The conclusion is well prepared and adequately discusses the results and conclusions obtained. It also shows the benefits of the results in improving the clinical management of the patient. |  |
| In the DISCUSSION section, the authors should mention the sampling limitation concerning the N of patients with AKI2-3 | Small sample size of AKI 2-3 group was added as a limitation to the discussion (Page 31). |
| Editorial and data presentation modifications |  |
| Table is misconfigured. | Table 1 modified (Pages 14-15) |
| Y axis of Figure 3E, correct 2β2M | Y-axis of figure 3E is correctly labeled as uβ2M. (Page 18) |
| Summary and general comments |  |
| The authors developed an important clinical toxin study involving Hump-nosed pit viper accidents in Sri Lanka: evaluation of plasma and urinary markers for prediction of Acute kidney injury. The results are solid and have an excellent number of markers, all very well suited and directly related to the AKI event. The authors noted that Cystatin C was an important biomarker in predicting a severe/moderate event within 4 hours of poisoning, giving credibility to its use in the clinical approach. The results obtained in the present study are of great clinical importance for Hump-nosed pit viper envenomations, hoping for an improvement in the clinical management of patients.  As a major weakness of the study, the population size of moderate/severe AKI patients did not provided enough credibility to the study. Suggestion is to gather the severity groups in just AKI patients. | Please refer to our response above stated under methods. |
